# Mitophagy in Yeast: Molecular Mechanism and Regulation

**DOI:** 10.3390/cells10123569

**Published:** 2021-12-17

**Authors:** Aleksei Innokentev, Tomotake Kanki

**Affiliations:** Department of Cellular Physiology, Niigata University Graduate School of Medical and Dental Sciences, Niigata 951-8510, Japan; alexei-g2@med.niigata-u.ac.jp

**Keywords:** Atg32, Atg43, autophagy, mitochondria, mitophagy, yeast

## Abstract

Mitophagy is a type of autophagy that selectively degrades mitochondria. Mitochondria, known as the “powerhouse of the cell”, supply the majority of the energy required by cells. During energy production, mitochondria produce reactive oxygen species (ROS) as byproducts. The ROS damage mitochondria, and the damaged mitochondria further produce mitochondrial ROS. The increased mitochondrial ROS damage cellular components, including mitochondria themselves, and leads to diverse pathologies. Accordingly, it is crucial to eliminate excessive or damaged mitochondria to maintain mitochondrial homeostasis, in which mitophagy is believed to play a major role. Recently, the molecular mechanism and physiological role of mitophagy have been vigorously studied in yeast and mammalian cells. In yeast, Atg32 and Atg43, mitochondrial outer membrane proteins, were identified as mitophagy receptors in budding yeast and fission yeast, respectively. Here we summarize the molecular mechanisms of mitophagy in yeast, as revealed by the analysis of Atg32 and Atg43, and review recent progress in our understanding of mitophagy induction and regulation in yeast.

## 1. Introduction

**Figure 1:**
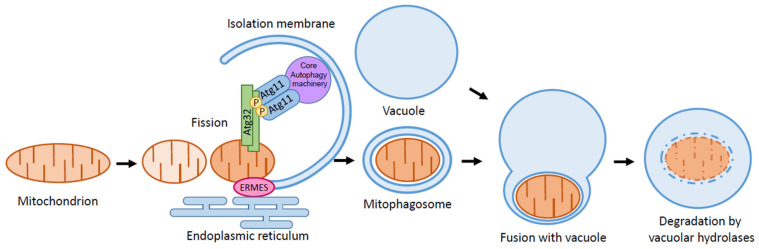
Schematic depiction of mitophagy. Mitophagy is a type of selective autophagy. Upon mitophagy induction, the mitophagy receptor Atg32 interacts with the adaptor protein Atg11. Atg11 then tethers the mitochondria to the PAS for selective sequestration by the isolation membrane. ERMES helps in expanding the isolation membrane to provide lipid sources from ER. Further extension of the isolation membrane leads to the formation of the mitophagosome, eventually fusing with the vacuole for the degradation of the mitochondria by vacuolar hydrolases.

Mitochondria are double-membrane-bound organelles that supply energy through the tricarboxylic acid cycle and oxidative phosphorylation (OXPHOS). Aside from this main role, mitochondria play many pivotal roles, such as their involvement in fatty acid oxidation, calcium buffering, amino acid synthesis, heme biosynthesis, and iron–sulfur cluster formation [1]. Mitochondria also regulate apoptosis and are involved in many signaling pathways [2]. Although reactive oxygen species (ROS) are natural byproducts of OXPHOS, excessive production of ROS leads to oxidative stress, which is harmful for cellular content, including the mitochondria themselves [3]. As superfluous or damaged mitochondria produce excessive ROS, this further damages mitochondria, resulting in further increased production of ROS. The excessive ROS eventually induce mitochondrial DNA mutation or deletion. Accumulation of damaged mitochondria leads to various diseases such as Alzheimer’s disease(AD), Parkinson’s disease, and myopathies and accelerates aging [4,5,6,7,8]. Thus, it is important for the cell to maintain mitochondrial homeostasis. Mitochondrial autophagy or mitophagy is one of the systems that maintain mitochondrial homeostasis (Figure 1). In yeast, mitophagy eliminates superfluous or damaged mitochondria and prevents the production of excessive ROS, thus contributing to mitochondrial homeostasis [9]. Mutations of the PARK2 and PARK6 genes, which encode Parkin and PINK1, respectively, cause autosomal recessive-juvenile Parkinsonism. It was suggested that Parkin and PINK1 accumulate on depolarized mitochondria and induce mitophagy to eliminate dysfunctional mitochondria [10]. It was recently reported that mitophagy is impaired in the hippocampus of AD patients. Mitophagy decreases the accumulation of insoluble amyloid-β and suppresses the hyperphosphorylation of tau protein in various AD models. Furthermore, stimulation of mitophagy could ease cognitive impairments on the AD model in *C. elegans* [11]. These findings suggest that manipulating mitophagy can be a promising way to treat diseases associated with mitochondrial dysfunction.

Autophagy is a highly conserved process of the degradation of cellular compartments that occurs in almost all eukaryotes. Autophagy was initially discovered in mammals in the 1960s [12,13,14], but the molecular mechanism and physiological role of autophagy have been largely unexplored until recently. The discovery of the autophagy-related (ATG) genes in yeast in the 1990s has rapidly advanced our understanding of autophagy. So far, 43 Atg proteins have been identified in yeast [15]. Induction of autophagy leads to the formation of the double-membranous structure termed the isolation membrane at the preautophagosomal structure or phagophore assembly site (PAS). This extends to sequester cytoplasmic components as cargos, and the isolation membrane closes and forms a vesicle called autophagosomes. Eventually, autophagosomes fuse with the vacuoles in yeast (lysosomes in mammals) to degrade the cargos by hydrolytic enzymes and recycling [16].

In contrast to the nonselective autophagy explained above, types of autophagy that selectively eliminate specific organelles and cellular compartments, such as mitochondria (mitophagy), endoplasmic reticulum (ER-phagy), peroxisomes (pexophagy), ribosomes (ribophagy), nuclei (nucleophagy), and the aminopeptidase 1 (Ape1) complex (cytoplasm-to-vacuole [Cvt]), exist [17,18,19,20,21,22]. Both selective and nonselective autophagy use the same machinery to form autophagosomes, and thus, the above process is thought to be common between selective and nonselective autophagy. Therefore, a unique feature of selective autophagy is how autophagic factors recognize cargos and how the isolation membrane incorporates them selectively.

## 2. Mitophagy Is A Selective Type of Autophagy

Autophagy was first thought to be the nonselective degradation of cytoplasmic compartments, and it was unclear whether or not mitochondria were degraded selectively by autophagy. In yeast, *Saccharomyces cerevisiae* (*S. cerevisiae*), Camougrand et al. first observed mitophagy using mitochondria-targeting fluorescent protein and identified *Uth1* as a mitophagy-related gene [23]. Although the involvement of *Uth1* in mitophagy has since been disproven [24], the observation of mitophagy using mitochondria-targeting fluorescent proteins is still a common method. Later, selective degradation of mitochondria by autophagy was suggested by showing that Atg11 is essential for mitophagy [25]. Atg11 had previously been known as an adaptor protein essential for other types of selective autophagy: the Cvt pathway and pexophagy [26,27]. Thus, the requirement of Atg11 for mitophagy suggests that this process is also a selective type of autophagy. Atg20 and Atg24 are members of the sorting nexin family of proteins and are required for the Cvt pathway and pexophagy but not for nonselective autophagy. It was shown that Atg20 and Atg24 were also required for mitophagy, again suggesting that mitophagy is a selective process [25,28].

## 3. Atg32 Is A Receptor for Mitophagy in *S. cerevisiae*

The cargo specific receptor is involved in selective autophagy in yeast. For the Cvt pathway, the cargo (the Ape1 complex) binds to the Atg19, a receptor to form the Cvt complex, and Atg19 interacts with the adaptor protein Atg11 [27]. Similarly, the pexophagy receptors, Atg30 in *Komagataella pastoris* and Atg36 in *S. cerevisiae*, localize on peroxisomes and interact with Atg11 upon pexophagy induction [29,30]. Atg11 accumulates on PAS by interacting with Atg1 and Atg9. As a result, Atg11 tethers the cargo to the PAS for the selective sequestration of the cargo by the isolation membrane. Mitophagy receptor Atg32, a 59 kDa, single-transmembrane protein that localizes on the outer membrane of the mitochondria, was identified by genome-wide screening for mitophagy-defective mutants in *S. cerevisiae* [25,28]. Atg32 is conserved among budding yeasts, such as *Candida glabrata* and *Pichia pastoris*, but not in fission yeasts [31]. Deletion of Atg32 leads to the complete inhibition of mitophagy but does not interfere with any other type of selective autophagy or nonselective autophagy. Atg32 interacts with Atg11 upon mitophagy induction, and Atg11 tethers the mitochondria to the PAS for the selective sequestration of the mitochondria by the isolation membrane (Figure 1).

When mitophagy is induced, Ser114 and Ser119 on Atg32 are phosphorylated, which is essential for the Atg32-Atg11 interaction and mitophagy [32]. Yeast two-hybrid analysis suggests that the N-terminal residues 51–150 of Atg32, which include the phosphorylation sites, interact with Atg11. In addition, recent studies revealed that amino acid residues 200–341 of Atg32 act as a pseudoreceiver that regulates the Atg32 interaction with Atg11 [33]. Furthermore, it was reported that Seh1-associated complex inhibiting TORC1 (SEACIT)-dependent TORC1 inactivation stabilizes the Atg32-Atg11 interaction [34]. Although it is known that Atg32 serves as a mitophagy-specific receptor protein, it is not yet clear how yeast selects dysfunctional mitochondria destined for mitophagy. It is speculated that phosphorylation of Pkp1 and Pkp2 (mitochondrial matrix-localized protein kinases) helps in distinguishing mitochondria destined for mitophagy [35].

## 4. The Role of the Atg32-Atg8 Interaction in Mitophagy

**Figure 2:**
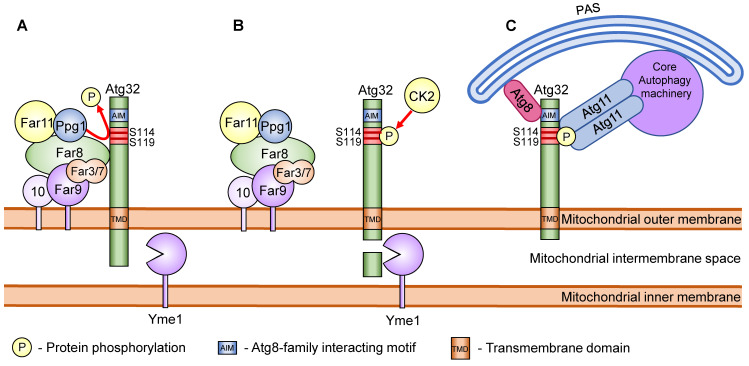
Schematic model of the regulation of Atg32 phosphorylation. (**A**) In the absence of mitophagy stimuli, phosphorylation of Atg32 is prevented by the Far complex and Ppg1. (**B**) Mitophagy stimuli lead to the dissociation of the Far complex from Atg32 and allow CK2 to phosphorylate Atg32 at Ser114 and Ser119. Mitophagy stimuli also lead to the proteolytic cleavage of the Atg32 C-terminal region by the i-AAA protease complex Yme1. (**C**) The phosphorylated Atg32 interacts with Atg11. Atg11 recruits the mitochondria to the PAS, where the core autophagy machineries initiate isolation membrane formation. Atg32 also interacts with the isolation membrane, localizing Atg8 via the AIM sequence and facilitating the extension of the isolation membrane along the surface of the mitochondria.

Most of the selective autophagy receptors have a W/Y-X-X-L/I/V sequence termed the Atg8 family interacting motif (AIM), where X can be any acidic amino acid [36]. Atg8 is conjugated to phosphatidylethanolamine (PE) and is localized to the isolation membrane. In selective autophagy, receptors are tethered to the isolation membrane via an AIM-Atg8 interaction that facilitates the selective sequestration of the cargo. Atg32 also has an AIM sequence that is used to interact with Atg8. Because the mutation of the AIM sequence of Atg32 only partially affects mitophagy, it is speculated that the Atg32-Atg8 interaction only facilitates the extension of the isolation membrane along the surface of the mitochondria [28] (Figure 2).

Additionally, it is speculated that the mitochondrial phosphatidylserine decarboxylase 1 (Psd1) recruits Atg8 to the mitochondria as Psd1 plays a particular role in mitophagy induced by nitrogen starvation. It is suggested that Psd1 provides PE for Atg8 lipidation and/or elongation of phagophore membrane, thus leading to recruitment of Atg8 to the mitochondria [37].

## 5. Atg43, A Mitophagy Receptor in *Schizosaccharomyces pombe*

Recently, mitophagy receptor Atg43 was identified by genome-wide screening for mitophagy-defective mutants in *S. pombe* [15]. Atg43 is a 27 kDa, single-transmembrane protein localized to the outer membrane of the mitochondria. Similar to Atg32 and other selective autophagy receptors, Atg43 has an AIM sequence that interacts with Atg8. Notably, in contrast to Atg32, the mutation of the AIM sequence on Atg43 completely suppresses mitophagy, suggesting that mitophagy in *S. pombe* is highly dependent on the AIM-mediated Atg43-Atg8 interaction. The AIM-dependent mitophagy process observed in *S. pombe* is consistent with mitophagy in mammals. Here, mitophagy receptors, such as BNIP3, NIX, FUNDC1, BCL2L13, and FKBP8, interact with the Atg8 mammalian homolog LC3 family proteins via an LC3-interacting region (LIR) on the receptors, where this interaction has been shown to be crucial for mitophagy [38,39,40,41,42].

## 6. Experimental Induction of Mitophagy in Yeast

Mitophagy can be induced in various ways. For example, mitophagy is induced by preculturing yeast in a medium with a nonfermentable carbon source and shifting it into a nitrogen starvation medium with a fermentable carbon source. In nonfermentable carbon source media, the cell obtains energy only from mitochondrial OXPHOS and promotes mitochondrial biogenesis. Then, in the nitrogen starvation medium with a fermentable carbon source, the cell begins obtaining energy from glycolysis and eliminates superfluous mitochondria via mitophagy. Another way to induce mitophagy is to culture yeasts in a medium with a nonfermentable carbon source until the stationary phase [43]. Mitophagy can be induced by depleting ethanol after respiratory growth in nonfermentable carbon source media [44]. Mitophagy can also be induced by rapamycin, a target of rapamycin (TOR) inhibitor. However, rapamycin also induces other types of autophagy, thus making it difficult to specifically separate results of mitophagy from those of bulk autophagy [45].

## 7. Regulation of Mitophagy in Yeast

Mitophagy is regulated at the transcriptional and posttranslational modification levels. Here, we discuss the mitophagy regulatory mechanism at each level.

### 7.1. Transcriptional Level

**Figure 3:**
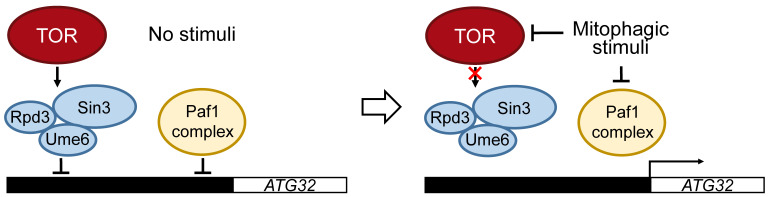
Regulation of *ATG32* transcription. In nutrient-rich conditions, transcription of *ATG32* is inhibited by Ume6–Sin3-Rpd3, which is regulated under TOR. The transcription of *ATG32* is also inhibited by the polymerase-associated factor 1 (Paf1) complex. Both the Ume6–Sin3-Rpd3 and Paf1 complexes directly bind to the promoter region of *ATG32* to inhibit the transcription of *ATG32*. Mitophagic stimuli, such as nitrogen starvation, inhibits TOR and releases Ume6–Sin3-Rpd3 and Paf1 complexes from the promoter region of *ATG32* to allow the transcription of *ATG32*.

It has been shown that transcription of *ATG32* is suppressed in nutrient-rich conditions and is induced by the inhibition of TOR by nutrient starvation or rapamycin treatment in *S. cerevisiae* and *K. pastoris.* The DNA-binding protein Ume6 and the histone deacetylase complex Sin3-Rpd3 directly interact with the promoter region of *ATG32* to repress its transcription. This repression of *ATG32* transcription by the Ume6–Sin3-Rpd3 complex is released by TOR inhibition [31] (Figure 3). It has also been shown that the polymerase-associated factor 1 (Paf1) complex represses *ATG32* and *ATG11* transcription in glucose-rich conditions. It is speculated that the Paf1 complex decreases *ATG32* transcription by binding to the *ATG32* promoter region and dissociates when yeast is exposed to glucose-starvation conditions [46,47] (Figure 3). It was reported that transcription of *ATG32* during starvation requires N-terminal acetyltransferase A (NatA), which consists of the catalytic subunit Ard1 and adaptor subunit Nat1. Deletion of NatA led to severe mitophagy defect due to a lack in Atg32 expression [48,49]. Furthermore, NatA contributes to the phosphorylation of Atg32 to promote mitophagy [48,49]. However, the precise mechanism of how NatA regulates mitophagy remains unclear.

An antioxidant, *N*-acetylcysteine (NAC), inhibits Atg32 expression and mitophagy [28,50]. Additionally, it was reported that there is a link between phospholipid biosynthesis and mitophagy. The deletion of the phospholipid methyl transferase Opi3 results in an increase in glutathione levels and suppresses the transcription of *ATG32* [51]. These findings suggest that oxidative stress induces the transcription of *ATG32* and mitophagy.

### 7.2. Posttranslational Level

The Atg32 protein is synthesized in the cytosol and is integrated into the mitochondrial outer membrane (MOM). It was recently reported that the Atg32 C-terminal domain was integrated into the MOM depending on the translocase of the outer membrane (TOM) receptor, Tom20. By contrast, the cytosolic N-domain of Atg32 greatly relied on the mitochondrial import complex (MIM) [52].

As previously discussed, mitophagy induction leads to phosphorylation of Atg32 at amino acid residues Ser114 and Ser119. This phosphorylation results in the interaction between Atg32 and Atg11. Importantly, the phosphorylation at Ser114 is more critical for mitophagy and the Atg32–Atg11 interaction than that at Ser119 [32]. The phosphorylation of Ser114 and Ser119 on Atg32 is mediated by casein kinase 2 (CK2). Thus the deletion of CK2 leads to a defect in mitophagy but not in other types of autophagy (Figure 2) [53]. Although CK2 is a constitutively active protein kinase, Atg32 is not phosphorylated when mitophagy is not induced. This suggests the presence of a mechanism to confront CK2 to prevent the phosphorylation of Atg32. Here, Ppg1 (PP2A-like protein phosphatase) and the Far complex (Far3, Far7, Far8, Far9, Far10, and Far11) were found to be negative regulators of Atg32 phosphorylation [54]. Briefly, the Far complex directly interacts with Atg32 and recruits Ppg1 to the Atg32 to dephosphorylate Atg32. Upon mitophagy induction, the Far complex dissociates from Atg32, allowing the phosphorylation of Atg32 by CK2 (Figure 2) [55]. Deletion of Ppg1 or one of the Far complex proteins (except for Far10) leads to constitutive phosphorylation of Atg32 even when mitophagy is not induced.

Mitogen-activated protein kinases (MAPKs), namely, Slt2 and Hog1, and the mitogen-activated protein kinase kinase, Pbs2, were reported to be required for mitophagy. Slt2 was required not only for mitophagy but also for pexophagy and influenced recruitment of mitochondria to the PAS [56]. On the other hand, Hog1 and Pbs2 were required for Atg32 phosphorylation [32,56]. Additionally, it was reported that the mitochondrial membrane phospholipid cardiolipin activates the MAPK pathway [57]. MAPK Bck1 and its signaling pathway were shown to be necessary for mitophagy induction. It is also speculated that mitophagy requires sensors of the ER and plasma membrane, such as Mid2, Mtl1, and the cell wall signaling proteins Wsc1, Wsc2, Wsc3, and Wsc4 [56,58].

Under mitophagy-inducing conditions, the C-terminal region of Atg32, located in the intermembrane space of the mitochondria, undergoes proteolytic processing [59]. The catalytic subunit of i-AAA protease complex Yme1 was found to be the mediator of the processing. Deletion of *YME1* or prevention of Atg32 processing by C-terminal tagging of Atg32 led to a defect in mitophagy and the disruption of the Atg32–Atg11 interaction (Figure 2). This suggests that Yme1 is important for the recruitment of the mitochondria to the PAS, but the factors that trigger this proteolytic modification remain unclear [24,59]. It was also reported that ubiquitination of at least Lys282 of Atg32 is necessary for Atg32 degradation by proteasomes [60].

Pep4 is a vacuolar protease that is required for maturation and activation of the other vacuolar hydrolases and thus essential for vacuolar hydrolase activity. Some Atg32 showed increased molecular weight by approximately 20 kDa in *pep4Δ* cells during starvation or rapamycin treatment. This suggests that the modification activates Atg32, and the activated Atg32 is delivered to the vacuole by mitophagy, whereas the inactive, nonmodified Atg32 is rapidly degraded on mitochondria by neither vacuolar proteases nor proteasome systems [61]. Knowledge of this nonmodified Atg32 degradation may be important for future inhibition of unnecessary mitochondrial degradation.

## 8. Other Factors That Regulate Mitophagy in Yeast

### 8.1. Mitochondria and Other Organelle Contact Sites

ER–mitochondria encounter structure (ERMES), a mediator for ER–mitochondria contacts in yeast, is necessary for mitophagy [62,63]. Artificial tethering of mitochondria and ER in ERMES deletion mutants rescues the mitophagy defect, suggesting that the ER–mitochondria contact facilitates mitophagy. In starvation conditions, ERMES is localized to isolation membrane expansion sites, suggesting that ERMES helps in expanding the isolation membrane by providing lipid sources from the ER (Figure 1). It is worth mentioning that the ubiquitination of the ERMES components, namely, Mdm34 and Mdm12, by E3 ligase Rsp5 facilitates efficient mitophagy [64].

In *Candida albicans*, Mcp1, a part of membrane contact sites called vacuole and mitochondrial patches (vCLAMPs), was reported to be necessary for mitophagy. The Mcp1 is a protein localized on both mitochondria and vacuole. *MCP1* deletion caused the accumulation of abnormally enlarged mitochondria due to failure in mitophagosome formation and corrupted stability of mitochondrial DNA [65].

### 8.2. Mitochondrial Dynamics

It is thought that mitochondrial fission separates the damaged portions of the mitochondria from the mitochondrial pool, and the separated mitochondria are eventually degraded by mitophagy. The fusion and fission of the mitochondria occur in response to various intracellular and extracellular stimuli [66,67]. It was shown that fission of mitochondria facilitates mitophagy by dividing mitochondria into small pieces that are easily engulfed by isolation membranes. Dnm1 (dynamin-related GTPase) accumulates at the site of the MOM Fis1 and Caf4/Mdv1 complex to divide the mitochondria. Deletion of any of these proteins led to reduced mitophagy [58,68,69]. When mitophagy is induced, mitochondrial fission machineries are recruited to the degrading mitochondria via the Atg11-Dnm1 interaction [68]. However, despite the involvement of mitochondrial fission in mitophagy, the elimination of the mitochondrial fission machinery does not completely block mitophagy [68,69,70,71].

### 8.3. Ubiquitination

The Ubp3-Bre5 deubiquitination complex is translocated to mitochondria during mitophagy in *S. cerevisiae*. This inhibits mitophagy and activates other types of autophagy, such as ribophagy, the Cvt pathway, and macroautophagy. This finding suggests that regulation of mitophagy via ubiquitination/deubiquitinating, common in mammals, exists also in yeast [20,72].

## 9. Conclusions

Mitophagy is a physiological process conserved in eukaryotic organisms, ranging from unicellular cellular organisms, like yeast, to multicellular organisms, such as mammals. In this review, we summarized the molecular mechanism and regulation of mitophagy in yeast. The key factor for mitophagy in *S. cerevisiae* is mitophagy receptor Atg32. The phosphorylation of Atg32, which is mediated by CK2 and is inhibited by the Ppg1–Far complex, triggers mitophagy. Thus, mitophagy is regulated by the balance of kinase and phosphatase. The question that remains is how the phosphorylation of Atg32 by the kinase and phosphatase is controlled and what the upstream signaling pathway connecting the mitophagy-inducing stimuli to Atg32 phosphorylation is. In addition, the mitophagy receptor Atg43 in *S. pombe* was recently identified. Here, Atg43 interacts with Atg8 via the AIM, and this interaction is crucial for mitophagy. One of the remaining questions is how the activity of Atg43 is regulated in *S. pombe*.

Recently, it was reported that Atg32 was required for spermidine synthesis via its involvement in the production of the spermidine precursor S-adenosylmethionine. Spermidine induces nitric oxide production during heat stress, which is necessary for cytoprotection [73]. It would be interesting to further uncover the connection between mitophagy, NO signaling, and polyamine synthesis.

Mitophagy has a vital role in mitochondrial homeostasis. Therefore, abnormalities in mitophagy are thought to be the cause of various diseases relating to mitochondrial dysfunction. The progression of our understanding of mitophagy in yeast is likely to open the door to further knowledge of diseases involving mitochondrial abnormalities.
